# Coastal grassland vegetation records from São Miguel Island (Azores) and the south-western coast of mainland Portugal

**DOI:** 10.3897/BDJ.13.e173529

**Published:** 2025-11-24

**Authors:** Hugo Renato M.G. Calado, António O. Soares, Ruben Heleno, Paulo A. V. Borges

**Affiliations:** 1 University of the Azores, cE3c – Centre for Ecology, Evolution and Environmental Changes/Azorean Biodiversity Group, CHANGE – Global Change and Sustainability Institute, Faculty of Science and Technology, Rua da Mãe de Deus, 9500-321 Ponta Delgada, São Miguel, Azores, Portugal University of the Azores, cE3c – Centre for Ecology, Evolution and Environmental Changes/Azorean Biodiversity Group, CHANGE – Global Change and Sustainability Institute, Faculty of Science and Technology, Rua da Mãe de Deus, 9500-321 Ponta Delgada São Miguel, Azores Portugal; 2 Centre for Functional Ecology, Associate Laboratory TERRA, Department of Life Sciences, University of Coimbra, Calçada Martim de Freitas, 3000-456, Coimbra, Portugal Centre for Functional Ecology, Associate Laboratory TERRA, Department of Life Sciences, University of Coimbra, Calçada Martim de Freitas, 3000-456 Coimbra Portugal; 3 University of Azores, CE3C—Centre for Ecology, Evolution and Environmental Changes, Azorean Biodiversity Group, CHANGE —Global Change and Sustainability Institute, School of Agricultural and Environmental Sciences, Rua Capitão João d’Ávila, Pico da Urze, 9700-042, Angra do Heroísmo, Azores, Portugal University of Azores, CE3C—Centre for Ecology, Evolution and Environmental Changes, Azorean Biodiversity Group, CHANGE —Global Change and Sustainability Institute, School of Agricultural and Environmental Sciences, Rua Capitão João d’Ávila, Pico da Urze, 9700-042 Angra do Heroísmo, Azores Portugal; 4 IUCN SSC Monitoring Specialist Group, Angra do Heroísmo, Azores, Portugal IUCN SSC Monitoring Specialist Group Angra do Heroísmo, Azores Portugal

**Keywords:** sampling event, occurrence, plants, Azores, mainland Portugal, coastal grasslands, photographic sampling

## Abstract

**Background:**

The present work provides an inventory of the plant species recorded in two distinct coastal grassland vegetations: the Azores Archipelago (São Miguel Island) and the south-western coast of mainland Portugal (Sesimbra and Sines Regions – Setúbal District). Sites were selected in both regions to have a similar general substrate (rocky), latitude and elevation. Thirty-one sites were selected in the coastal grasslands: thirteen were located on São Miguel Island and eighteen on the mainland, distributed across Sesimbra (12) and Sines (6). All sites were visited once during the spring of 2022. In each site, 30 photos were taken at 5-metre intervals, for a total of 930 photos.

The Sesimbra and Sines Regions were chosen because they represent well-preserved examples of coastal grassland vegetation on the south-western coast of mainland Portugal, sharing similar environmental characteristics with the coastal grasslands of São Miguel Island, such as rocky substrate, Atlantic exposure and comparable latitude. This design allows a meaningful comparison between insular and continental vegetation under similar abiotic conditions, providing a standardised framework for documenting coastal plant diversity across contrasting geographic contexts.

**New information:**

Most records consolidate previously documented occurrences from national repositories, but here they are re-validated through standardised, plot-based, georeferenced surveys that harmonise taxonomy and metadata. Crucially, this release constitutes the first coordinated, standardised inventory of coastal grassland vascular plants spanning the Azores and the south-western mainland of Portugal, enabling direct cross-regional comparisons. In addition to several within-plot distributional updates, we document multiple introduced and invasive taxa, most notably *Carpobrotus edulis* and *Oxalis pes-caprae*, that are known to reshape coastal communities. As expected for Atlantic coastal grasslands, the flora is dominated by Asteraceae, Poaceae, Fabaceae and Brassicaceae, providing a baseline for long-term monitoring and management across both archipelagic and continental shores.

## Introduction

Grasslands, including coastal variants, have been widely studied in terms of plant and faunal composition (Jones and Donnelly 2004, Feher et al. 2021). These ecosystems are of particular importance because they serve as habitats for numerous organisms that provide crucial ecosystem services for humans, such as nutrient cycling, carbon sequestration and pollination (Peters et al. 2016). Nevertheless, studies focusing specifically on coastal grassland vegetation remain comparatively scarce, leaving much to be explored (Calado et al. 2024).

Vegetation cover plays a key role in the structuring of ecosystems, with certain organisms being closely linked to the type of vegetation present, without which they cannot establish themselves. Examples include the monarch butterfly (*Danaus plexippus* (Linnaeus, 1758)) and plants of the genus *Asclepias* spp. (Greenstein et al. 2022), as well as the “Lundy cabbage flea beetle” (*Psylliodes luridipennis*, Chrysomelidae), which depends on the plant *Coincya wrightii*, endemic to Lundy Island, England (Compton et al. 2007). The decline of these plant populations would certainly lead to a reduction of the respective arthropod communities.

Anthropogenic activities and climate change significantly affect the structure of natural ecosystems (Wick et al. 2016, Lister and Garcia 2018, Bardgett et al. 2021, Dai et al. 2022). Oceanic islands are particularly sensitive to such pressures, as their restricted area and isolation make them vulnerable to trade, tourism and biological invasions (Delgado and Riera 2020, Borges et al. 2022, Boieiro et al. 2024, Calado et al. 2025b). Understanding the composition of coastal vegetation is, therefore, essential to assess how such factors influence ecosystem structure and biodiversity.

Coastal grassland vegetation represents a valuable component of Atlantic ecosystems, combining Euro-Atlantic, Mediterranean and Macaronesian floristic elements. Understanding its composition across island and mainland contexts provides insights into biogeographical patterns, habitat specificity and potential drivers of species turnover.

This data paper aims to document and make publicly available a standardised inventory of plant species recorded in coastal grasslands of São Miguel Island (Azores) and the Sesimbra and Sines Regions (Setúbal District, south-western coast of mainland Portugal). The dataset constitutes a baseline for future ecological and biogeographical studies, biodiversity monitoring and conservation planning.

## General description

### Purpose

The primary objective of this study is to provide a comprehensive inventory of plants in the coastal grasslands across São Miguel Island (Azores Archipelago), Sesimbra and Sines Regions (Setúbal District in south-western coast of mainland Portugal).

### Additional information

The dataset includes information on the diversity and composition of plants communities, recorded through the project's monitoring surveys.

## Project description

### Title

Phenotypic Plasticity of Pest and Biological Control Agents: Contrasting Mainland and Insular Coastal Ecosystems

### Personnel

Paulo A.V. Borges, Hugo Calado, Ruben Heleno, António O. Soares, Lurdes Borges.

### Study area description

**Figure 1.**
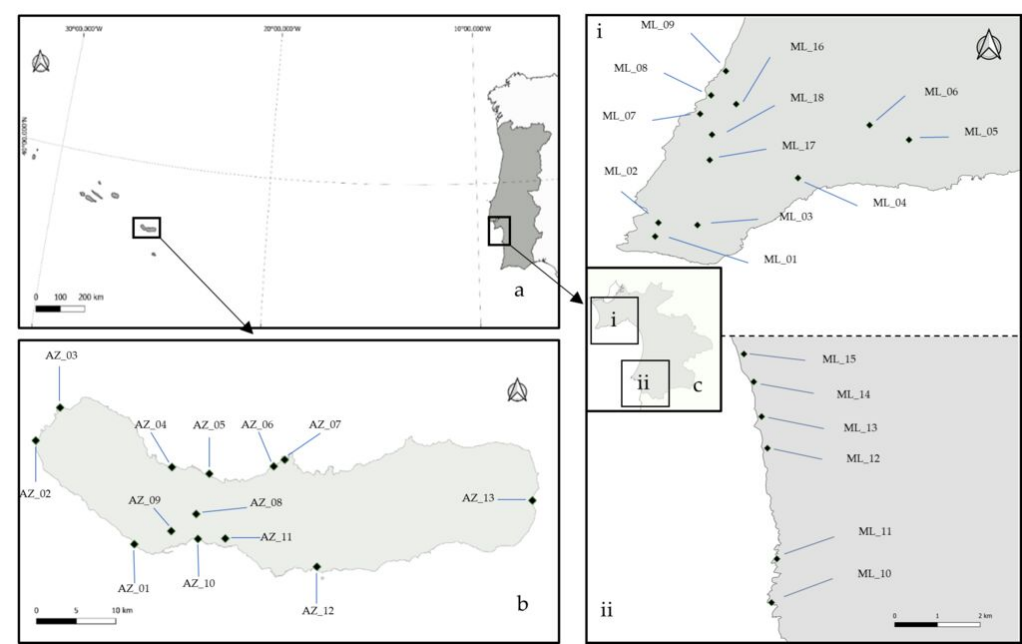
Sampling areas with the plots indicated: **a** Azores Archipelago and Portugal mainland; **b** São Miguel’s Island; **c** Setúbal District; (**i**) Sesimbra; (**ii**) Sines (source: Calado et al. (2024)).

The study was conducted in the coastal grasslands of São Miguel Island (Azores Archipelago, North Atlantic) and south-western coast of mainland Portugal (Setúbal District – Sesimbra and Sines Regions) (Fig. 1).

The Azores Archipelago is located in the centre of the North Atlantic, approximately 1,600 km from mainland Portugal, extending about 600 km between Santa Maria and Corvo (37°–40° N; 25°–31° W). The Archipelago has regular and abundant rainfall, an oceanic climate with relatively stable temperatures, high humidity throughout the year and persistent winds, especially during autumn and winter (Santos et al. 2004; Borges et al. 2019; IPMA 2020).

Mainland Portugal is located in south-western Europe (33°–43° N; 9°–6° W), with a total land area of approximately 89,015 km². The Setúbal District lies on the south-western coast of mainland Portugal, south of Lisbon and encompasses the regions of Sesimbra and Sines. The mainland has a Mediterranean climate, characterised by warm, dry summers and cool, wet winters (Carvalho et al. 2014; de Lima et al. 2015).

### Design description

Thirty-one plots were selected, with similar general substrate (rocky), latitude and elevation, in coastal grasslands in both ecosystems: The Azores Archipelago and Portugal mainland.

### Funding

H.R.M.G.C. was funded by the Regional FRCT Ph.D. Grant M3.1.a/F/012/2021: Phenotypic Plasticity of Pest and Biological Control Agents: Contrasting Mainland and Insular Coastal Ecosystems. A.O.S. and P.A.V.B. were also funded by the projects Pluriannual Funding FCT-UIDB/00329/2020-2024 - DOI 10.54499/UIDB/00329/2020 (Thematic Line 1 – integrated ecological assessment of environmental change on biodiversity), Azores DRCT Pluriannual Funding (M1.1.A/FUNC.UI&D/010/2021-2024) and PAVB by the project AZORESBIOPORTAL – PORBIOTA (ACORES-01-0145 FEDER-000072). and by the Regional Directorate for Science, Innovation and Development [Regional Government of the Azores] through the PROSCIENTIA Incentive System M1.1.A/FUNC.UI&D/021/2025 [UI&D/GBA/2025].

## Sampling methods

### Study extent

The study was conducted in the coastal grasslands of São Miguel Island (Azores Archipelago, North Atlantic) and south-western coast of mainland Portugal (Setúbal District – Sesimbra and Sines Regions).

### Sampling description

All 31 sites were visited once during the spring of 2022 (March–May), resulting in 31 sampling events. At each site, a 45-minute survey was carried out to record vegetation composition: 30 minutes were dedicated to photographic sampling and 15 minutes to direct visual observation and preliminary species identification. Each site covered an area of approximately 2500 m², defined for methodological consistency rather than by minimum area calculation, to ensure adequate spatial coverage and logistical feasibility across all sampling locations.

Thirty photographs were taken per site, at 5-metre intervals and 1.2 metres above ground level, totalling 930 photographs. Photographs were taken using a Sony HD Movie 720p camera. No vegetation cover-abundance scale (e.g. Braun–Blanquet) was applied, as the study focused exclusively on documenting species occurrences rather than quantitative vegetation analysis.

### Quality control

**Figure 2.**
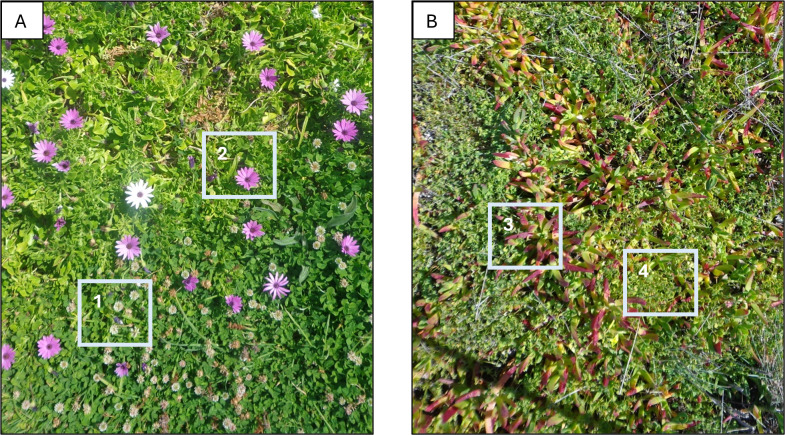
Examples of ground photos from different plots in the Azores (A) and mainland Portugal (B), showing some of the plant species and morphospecies: 1 – *Dimorphotheca fruticosa*; 2 – *Trifolium* sp.; 3 – *Carpobrotus edulis*; 4 – *Oxalis pes-caprae*.

All photographs were later examined individually on a computer to verify and confirm species identifications (Fig. 2). Photographs were then organised into a digital archive to facilitate taxonomic validation.

When necessary, field observations and photographs were cross-checked against online resources, such as the Azorean Biodiversity Portal and the Flora-On database, to ensure taxonomic consistency.

### Step description

In the laboratory, all photographs were organised and used for taxonomic identification. Plant taxa were identified to the species level whenever possible, using the Azorean Biodiversity Portal and the Flora-On database as references. When reproductive structures were absent, morphologically distinct plant entities were classified as morphospecies, following the same operational criteria applied in previous arthropod studies (Calado et al. 2024). Each morphospecies represents a unique morphological unit and is treated as an Operational Taxonomic Unit (OTU).

Photographic data were obtained using a Sony HD Movie 720p camera, with 30 photographs taken per site at 5-metre intervals and 1.2 metres above ground level, totalling 930 images. This equipment provided sufficient image resolution for accurate post-field identification of plant taxa and morphospecies.

## Geographic coverage

### Description

**General spatial coverage**: The study was conducted in the coastal grasslands of São Miguel Island (Azores Archipelago, North Atlantic) and mainland Portugal (Setúbal District – Sesimbra and Sines Regions).

### Coordinates

37°43'7''N and 38°27'43''N Latitude; 25°51'12''W and 8°47'36''W Longitude.

## Taxonomic coverage

### Description

**Taxonomic ranks**

Kingdom: Plantae

Phylum: Magnoliophyta, Pinophyta, Pteridophyta.

Class: Genopsida, Liliopsida, Magnoliopsida, Pinopsida, Polypodiopsida.

Order: Alismatales, Apiales, Asparagales, Asterales, Boraginales, Brassicales, Caryophyllales, Dipsacales, Ephedrales, Ericales, Fabales, Fagales, Gentianales, Geraniales, Lamiales, Malpighiales, Malvales, Myrtales, Oxalidales, Pinales, Poales, Polypodiales, Proteales, Ranunculales, Rosales, Sapindales, Saxifragales, Solanales, Vitales, Zingiberales.

Family: Acanthaceae, Agavaceae, Aizoaceae, Amaranthaceae, Amaryllidaceae, Anacardiaceae, Apiaceae, Apocynaceae, Araceae, Asparagaceae, Asphodelaceae, Aspleniaceae, Asteraceae, Boraginaceae, Brassicaceae, Cactaceae, Campanulaceae, Caryophyllaceae, Crassulaceae, Cupressaceae, Dennstaedtiaceae, Dryopteridaceae, Ephedraceae, Ericaceae, Euphorbiaceae, Fabaceae, Fagaceae, Geraniaceae, Juncaceae, Lamiaceae, Linaceae, Malvaceae, Myrtaceae, Oleaceae, Onagraceae, Orchidaceae, Orobanchaceae, Oxalidaceae, Papaveraceae, Pinaceae, Pittosporaceae, Plantaginaceae, Plumbaginaceae, Poaceae, Polygonaceae, Primulaceae, Proteaceae, Rhamnaceae, Rosaceae, Rubiaceae, Scrophulariaceae, Solanaceae, Tamaricaceae, Thymelaeaceae, Tropaeolaceae, Urticaceae, Valerianaceae, Verbenaceae, Vitaceae, Zingiberaceae.

## Temporal coverage

### Notes

3 March 2022 – 17 May 2022.

## Collection data

### Collection name

Renato_PhD

### Collection identifier

PHEPLA

### Specimen preservation method

Photo Record

## Usage licence

### Usage licence

Creative Commons Public Domain Waiver (CC-Zero)

## Data resources

### Data package title

Coastal Grassland Vegetation Records from São Miguel Island (Azores) and Mainland Portugal

### Resource link

https://ipt.gbif.pt/ipt/resource?r=coastal_plants

### Alternative identifiers

https://www.gbif.org/dataset/2265c6e2-4ab7-4af2-93af-ba377a7d4e43

### Number of data sets

2

### Data set 1.

#### Data set name

Event Table

#### Data format

Darwin Core Archive

#### Character set

UTF-8

#### Download URL

http://ipt.gbif.pt/ipt/resource?r=coastal_plants

#### Data format version

1.2

#### Description

The dataset was published in the Global Biodiversity Information Facility platform, GBIF (Calado et al. 2025a). The following data table includes all the records for which a taxonomic identification of the species was possible. The dataset submitted to GBIF is structured as a sample occurrence dataset that has been published as a Darwin Core Archive (DwCA), which is a standardised format for sharing biodiversity data as a set of one or more data tables. The core data file contains 31 records (eventID). This GBIF IPT (Integrated Publishing Toolkit, Version 2.5.6) archives the data and, thus, serves as the data repository. The data and resource metadata are available for download in the Portuguese GBIF Portal IPT.

**Data set 1.** 

Column label	Column description
eventID	Identifier of the events, unique for the dataset.
locationID	Identifier of the locations, unique for the dataset.
country	The name of the country or major administrative unit in which the Location occurs (Portugal).
countryCode	The standard code for the country in which the Location occurs (PT).
stateProvince	The name of the next smaller administrative region than country (state, province, canton, department, region etc.) in which the Location occurs.
county	The full, unabbreviated name of the next smaller administrative region than stateProvince (county, shire, department etc.) in which the Location occurs.
municipality	The full, unabbreviated name of the next smaller administrative region than county (city, municipality etc.) in which the Location occurs.
locality	The specific description of the place.
verbatimLocality	The original textual description of the place.
locationRemarks	Comments or notes about the Location.
habitat	The habitat for an Event (coastal grasslands).
minimumElevationInMetres	The lower limit of the range of elevation (altitude, usually above sea level), in metres.
decimalLatitude	Approximate centre point decimal latitude of the field site in GPS coordinates.
decimalLongitude	Approximate centre point decimal longitude of the field site in GPS coordinates.
geodeticDatum	Standard Global Positioning System coordinate reference for the location of the sample collection points.
coordinateUncertaintyinMetres	Uncertain value of coordinate metrics.
coordinatePrecision	Value in decimal degrees to a precision of six decimal places.
georeferenceSources	Navigation system used to record the location of sample collections.
samplingProtocol	The sampling protocol used to capture the species (45 minutes at an area of 2500 m^2^; 30 minutes taking photos, 15 minutes identifiing plants).
sampleSizeValue	A numeric value for a measurement of the size (time duration, length, area or volume) of a sample in a sampling Event.
sampleSizeUnit	The unit of measurement of the size (time duration, length, area or volume) of a sample in a sampling Event.
samplingEffort	The amount of effort expended during an Event (1 person).
year	Year the sample was collected (2022).
month	The integer month in which the Event occurred.
day	The integer day of the month on which the Event occurred.
eventDate	The date-time or interval during which an Event occurred.

### Data set 2.

#### Data set name

Occurrence Table

#### Data format

Darwin Core Archive

#### Character set

UTF-8

#### Download URL

http://ipt.gbif.pt/ipt/resource?r=coastal_plants

#### Data format version

1.2

#### Description

The dataset was published in the Global Biodiversity Information Facility platform, GBIF (Calado et al. 2025a). The following data table includes all the records for which a taxonomic identification of the species was possible. The dataset submitted to GBIF is structured as an occurrence table that has been published as a Darwin Core Archive (DwCA), which is a standardised format for sharing biodiversity data as a set of one or more data tables. The core data file contains 742 records (occurrenceID). This GBIF IPT (Integrated Publishing Toolkit, Version 2.5.6) archives the data and, thus, serves as the data repository. The data and resource metadata are available for download in the Portuguese GBIF Portal IPT.

**Data set 2.** 

Column label	Column description
eventID	Identifier of the events, unique for the dataset.
type	Type of the record, as defined by the Dublin Core Standard.
licence	Reference to the licence under which the record is published.
institutionID	The identity of the institution publishing the data.
collectionID	The identity of the collection publishing the data.
institutionCode	The code of the institution publishing the data (UAc).
collectionCode	The code of the collection where the specimens are conserved (PHEPLA).
datasetName	Name of the dataset (Renato_PhD).
basisOfRecord	The nature of the data record.
recordedBy	A list (concatenated and separated) of names of people, groups or organisations who performed the sampling in the field.
occurrenceID	Identifier of the record, coded as a global unique identifier.
kingdom	Kingdom name.
phylum	Phylum name.
class	Class name.
order	Order name.
family	Family name.
genus	Genus name.
specificEpithet	Specific epithet name.
infraspecificEpithet	Infraspecific epithet name.
scientificNameAuthorship	The authorship information for the scientificName formatted according to the conventions of the applicable nomenclaturalCode.
identificationRemarks	Comments or notes about the Identification (Morphospecie's number in Renato PhD Collection).
identifiedBy	A list (concatenated and separated) of names of people, groups or organisations who assigned the Taxon to the subject.
dateIdentified	The date on which the subject was determined as representing the Taxon.
scientificName	The full scientific name, with authorship and date information if known.
taxonRank	Lowest taxonomic rank of the record.

## Additional information

**Figure 3.**
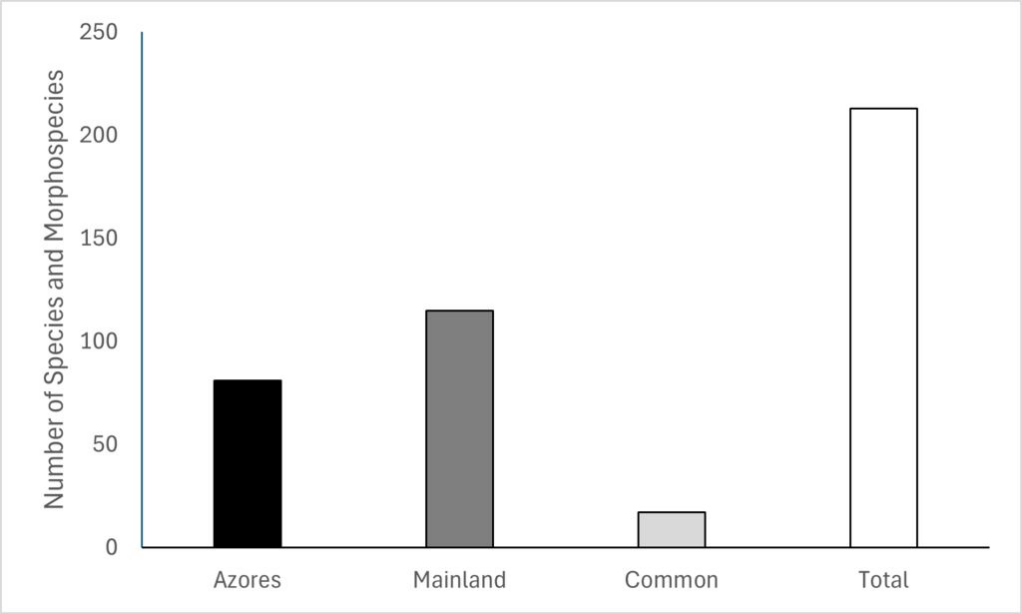
Number of species and morphospecies collected in the Azores and in the Portugal mainland.

A total of 213 plants species or morphospecies were recorded in the two coastal grasslands (Azores = 98; Mainland = 132). In the Azores, 98 species and morphospecies belonging to three classes were collected: Liliopsida, Magnoliopsida and Polypodiopsida. In the Mainland, a total of 132 species and morphospecies were collected, belonging to four classes: Genopsida, Liliopsida, Magnoliopsida and Pinopsida. In total, 17 species were common between the two regions (Fig. 3) (Table 1).

### Conclusions

This data paper provides a standardised, georeferenced inventory of plant species recorded in coastal grasslands of São Miguel Island (Azores Archipelago) and in the Sesimbra and Sines Regions (Setúbal District, in the south-western coast of mainland Portugal). A total of 213 taxa were recorded, including 17 species common to both regions. The dataset reveals higher species richness in mainland coastal grasslands compared with those of São Miguel Island.

Two invasive species, *Carpobrotus edulis* and *Oxalis pes-caprae*, were documented in both regions. No new endemic or threatened species were identified, although the dataset updates and validates existing distributional records.

The dataset, already accessible through GBIF (Calado et al. 2025a), is presented here in a validated and standardised format to ensure transparency, interoperability and long-term usability. By providing detailed methodological, taxonomic and metadata documentation, this data paper supports future ecological and biogeographical research on Atlantic coastal ecosystems (Boieiro et al. 2024; Calado et al. 2025b), as well as biodiversity monitoring and conservation planning across these habitats.

## Figures and Tables

**Table 1.** Common species in the Azores and mainland coastal grasslands.

**Class**	**Order**	**Family**	**Species**
Liliopsida	Poales	Poaceae	*Arundo donax* L.
Magnoliopsida	Caryophyllales	Aizoaceae	*Carpobrotus edulis* (L.) N.E.Br.
	Apiales	Apiaceae	*Foeniculum vulgare* Mill.
			*Daucus carota* L.
	Asterales	Asteraceae	*Galactites tomentosus* Moench
			*Coleostephus myconis* (L.) Rchb.f.
			*Sonchus tenerrimus* L.
	Brassicales	Brassicaceae	*Lobularia maritima* (L.) Desv.
	Fabales	Fabaceae	*Ornithopus compressus* L.
	Oxalidales	Oxalidaceae	*Oxalis pes-caprae* Schreb.
	Lamiales	Plantaginaceae	*Plantago lanceolata* L.
			*Plantago coronopus* L.
			*Veronica persica* Poir.
	Ericales	Primulaceae	*Lysimachia arvensis* (L.) U.Manns & Anderb.
	Gentianales	Rubiaceae	*Sherardia arvensis* L.
	Solanales	Solanaceae	*Solanum mauritianum* Scop.
	Rosales	Urticaceae	*Parietaria judaica* L.
